# Towards automated video-based assessment of dystonia in dyskinetic cerebral palsy: A novel approach using markerless motion tracking and machine learning

**DOI:** 10.3389/frobt.2023.1108114

**Published:** 2023-03-02

**Authors:** Helga Haberfehlner, Shankara S. van de Ven, Sven A. van der Burg, Florian Huber, Sonja Georgievska, Ignazio Aleo, Jaap Harlaar, Laura A. Bonouvrié, Marjolein M. van der Krogt, Annemieke I. Buizer

**Affiliations:** ^1^ Amsterdam UMC location Vrije Universiteit Amsterdam, Rehabilitation Medicine, Amsterdam, Netherlands; ^2^ Amsterdam Movement Sciences, Rehabilitation and Development, Amsterdam, Netherlands; ^3^ Department of Rehabilitation Sciences, Katholieke Universiteit Leuven, Campus Bruges, Bruges, Belgium; ^4^ Netherlands eScience Center, Amsterdam, Netherlands; ^5^ Centre for Digitalization and Digitality, University of Applied Sciences Düsseldorf, Düsseldorf, Germany; ^6^ Moveshelf Labs B.V., Utrecht, Netherlands; ^7^ Department Biomechanical Engineering, Delft University of Technology (TU Delft), Delft, Netherlands; ^8^ Emma Children’s Hospital, Amsterdam UMC, Amsterdam, Netherlands

**Keywords:** cerebral palsy, movement disorders, machine learning, motion capture, markerless skeleton tracking, human pose estimation

## Abstract

**Introduction:** Video-based clinical rating plays an important role in assessing dystonia and monitoring the effect of treatment in dyskinetic cerebral palsy (CP). However, evaluation by clinicians is time-consuming, and the quality of rating is dependent on experience. The aim of the current study is to provide a proof-of-concept for a machine learning approach to automatically assess scoring of dystonia using 2D stick figures extracted from videos. Model performance was compared to human performance.

**Methods:** A total of 187 video sequences of 34 individuals with dyskinetic CP (8–23 years, all non-ambulatory) were filmed at rest during lying and supported sitting. Videos were scored by three raters according to the Dyskinesia Impairment Scale (DIS) for arm and leg dystonia (normalized scores ranging from 0–1). Coordinates in pixels of the left and right wrist, elbow, shoulder, hip, knee and ankle were extracted using DeepLabCut, an open source toolbox that builds on a pose estimation algorithm. Within a subset, tracking accuracy was assessed for a pretrained human model and for models trained with an increasing number of manually labeled frames. The mean absolute error (MAE) between DeepLabCut’s prediction of the position of body points and manual labels was calculated. Subsequently, movement and position features were calculated from extracted body point coordinates. These features were fed into a Random Forest Regressor to train a model to predict the clinical scores. The model performance trained with data from one rater evaluated by MAEs (model-rater) was compared to inter-rater accuracy.

**Results:** A tracking accuracy of 4.5 pixels (approximately 1.5 cm) could be achieved by adding 15–20 manually labeled frames per video. The MAEs for the trained models ranged from 0.21 ± 0.15 for arm dystonia to 0.14 ± 0.10 for leg dystonia (normalized DIS scores). The inter-rater MAEs were 0.21 ± 0.22 and 0.16 ± 0.20, respectively.

**Conclusion:** This proof-of-concept study shows the potential of using stick figures extracted from common videos in a machine learning approach to automatically assess dystonia. Sufficient tracking accuracy can be reached by manually adding labels within 15–20 frames per video. With a relatively small data set, it is possible to train a model that can automatically assess dystonia with a performance comparable to human scoring.

## 1 Introduction

Videos have been used to visually assess neurologic movement disorders for more than 100 years (Aubert, 2002; Barboi et al., 2004; Reynolds et al., 2011). They have shown great value in diagnostics, documentation of disease progression, and evaluation of treatment outcome (Sambati et al., 2019). Standardized video protocols have been established for clinical rating scales, such as the Unified Dyskinesia Rating Scale for Parkinson’s disease (Goetz et al., 2008) and the Dyskinesia Impairment Scale (DIS) in dyskinetic cerebral palsy (CP) (Monbaliu et al., 2012). With the widespread use of smartphones, self-recorded home videos have also shown their clinical value (Billnitzer and Jankovic, 2021).

However, a major drawback of using videos in the evaluation of movement disorders is that videos must be assessed by a clinician, and evaluation is dependent on training and experience and remains subjective. In addition, accurate rating of videos is time-consuming. Open sharing of video data for training and alignment of scoring between centers is difficult on a large scale due to privacy issues. Recently, there has been a rapidly emerging field within computer vision using algorithms to automatically detect actions within skeleton stick figures (e.g., surveillance (Lin et al., 2021) or emotion recognition (Shi et al., 2020)).

The potential of these techniques for a clinical purpose within CP has been shown recently for the early detection of CP in infants at risk (Groos et al., 2022) or the predicting of gait parameters from common videos in ambulatory children with CP (Kidzinski et al., 2020). To our knowledge, automated video-based assessment has not yet been applied to complex movement disorders, such as dystonia in dyskinetic CP (Haberfehlner et al., 2020).

Dyskinetic CP has a prevalence of approximately 0.12–0.3 in every 1000 live births in Europe (Himmelmann et al., 2009). This group of children and adults generally experience severe limitations in mobility, manual ability and communication (Monbaliu et al., 2017a). The dyskinetic movements and postures are characterized by two features: (1) dystonia, described by abnormal patterns of posture and/or slow movements, and (2) choreoathetosis, characterized by faster involuntary, uncontrolled, recurring and occasionally stereotyped movements (Monbaliu et al., 2017a). Dystonia and choreoathetosis can both occur during rest and activity (Monbaliu et al., 2012). Although dystonia and choreoathetosis often coexist in the same patient, dystonia is predominant (Monbaliu et al., 2016) and most strongly linked to daily life limitations and quality of life (Monbaliu et al., 2017b).

Interventions in this group are mostly aimed at reducing dystonia and include invasive neuromodulation treatments such as intrathecal baclofen (Bonouvrie et al., 2019) and deep brain stimulation (Koy and Timmermann, 2017). In addition, advanced rehabilitation techniques (such as alternative computer access solutions and powered mobility (Bekteshi et al., 2020)) are commonly applied within this group. Frequent and effective monitoring of dystonia would be extremely important for the indication and evaluation of these interventions but is often not applied clinically due to time constraints and lack of objectiveness in quantifying severity.

This paper proposes a new way to score dystonia in dyskinetic CP by using data extracted by markerless motion tracking from videos (i.e., x, y coordinates of body parts) and use supervised machine learning to predict dystonia scores from computed movement and position features from these extracted x, y coordinates. Such a data-driven approach solely from video recordings has the potential to improve in the future if more data come available and would offer a cost-effective, easily executable and accessible way to evaluate dystonia.

Markerless motion tracking using single camera recordings is a rapidly evolving technology. Recently developed open-source toolbox codes (e.g., DeeperCut (Insafutdinov et al., 2016), OpenPose (Cao et al., 2017) and PoseNet (Oved et al., 2018)) allow users fast (or even real-time (Oved et al., 2018)) human pose estimation based on convolutional neural networks. DeepLabCut (Mathis et al., 2018; Nath et al., 2019) is based on DeeperCut, but has been tailored for use in different environments and for user-defined (body) landmarks using transfer learning with relatively small amounts of manually labelled data. DeepLabCut can be used in combination with pre-trained human models on the MPII Human Pose Dataset (Andriluka et al., 2014a; 2014b; Insafutdinov et al., 2016). DeepLabCut seem to be a promising tool to apply to videos of children and young adults with dyskinetic CP.

Within different fields of movement analysis machine learning/deep learning techniques are increasingly used to monitor, automatically recognize activities or (pathological) movements or evaluate training or treatment outcome, up to now mainly using manually crafted features, based on domain knowledge, as input (Halilaj et al., 2018; Khera and Kumar, 2020; Dorschky et al., 2023).

To demonstrate proof-of-concept of the proposed method, we aimed to assess whether the automated video-based method performs equally well to human performance (i.e., compare inter-rater to model-rater accuracy). In addition, the accuracy of automatically tracking body landmarks from 2D videos compared to human labeling in children and young adults with dyskinetic CP was assessed. Therefore, the study consists of two steps: (1) assessment of tracking accuracy and (2) the development of prediction models for an automated method for dystonia assessment.

## 2 Materials and methods

### 2.1 Participants

A total of 187 videos of 34 unique individuals with dyskinetic CP who participated in a randomized placebo‐controlled trial on the effects of intrathecal baclofen (IDYS trial, Dutch Trial Register NTR3642) (Bonouvrie et al., 2019; Bonouvrie et al., 2022) from Amsterdam University Medical Centers, location VUmc, were used for the current analysis. Videos were recorded at baseline, 3-month follow-up and 12-month follow-up. The inclusion criteria for the IDYS trial were as follows: (1) presenting with dyskinetic CP; (2) classified at Gross Motor Function Classification System (GMFCS) (Palisano et al., 2008) levels IV and V (i.e. non-walking); (3) aged 4–25 years; (4) lesions on magnetic resonance imaging; and (5) eligible for intrathecal baclofen treatment using commonly applied criteria. Patients at baseline had the following characteristics: age 14.0 ± 3.9 (mean ± standard deviation) years; weight: 32.7 ± 11.8 kg; height: 147.1 ± 20.4 cm; 8 females/26 males; Gross Motor Function Classification System (GMFCS) IV (*n* = 13) or V (*n* = 20), Manual Ability Classification System (MACS) (Eliasson et al., 2006): III (*n* = 3), IV (*n* = 8), V (*n* = 22). This secondary analysis of the video data was approved by the local medical ethics committee.

### 2.2 Videos

Videos were recorded as part of the DIS to clinically evaluate leg and arm dystonia (Monbaliu et al., 2012). The items “lying in rest” and “sitting in rest” were used for the current analysis. These positions are commonly used to assess non-ambulatory individuals with dyskinetic CP. Participants were asked during these items to sit and lie quiet, without intentional movements. Videos were recorded at 25 Hz. Within the sitting videos all sequences were 20 s (500 frames) long. Within the sitting videos all sequences were 20 s (500 frames) long. Within the lying videos the majority (86 videos out of 94) were between 19 and 20 s (475–500 frames), three video sequences had a length between 10 and 19 s (250–474 frames) and eight video sequences had a length between 4 and 10 s (100–249 frames). Within all videos, the faces of the children and caregivers were blurred. Subsequently, the videos were all converted to the same size (720 width x 575 height pixels, which covered an area of approximately 3 × 2 meters, yielding an image resolution of 0.4 cm per pixel) and the same video format (avi, x264 codec) using Any Video Converter (version 5.7.8, Anvsoft Inc.).

### 2.3 Dataset assessment of tracking accuracy

For the assessment of tracking accuracy, only the videos in which children were lying in rest on a mat (*n* = 33, 94 videos) were selected. This is a position that enables this group of non-walking individuals to be assessed without external support. This position deviates from standard positions within the training data of the pretrained human model (Andriluka et al., 2014a). For tracking accuracy, the data were split into a development set and a generalization set. 80% of the participants (i.e., a total of 27 subjects with 76 related videos were randomly placed in the development set, and 20% of participants (i.e., six participants with 18 related videos) in the generalization set. The development set was used to train models with an increasing number of manually labeled frames (as explained in detail below). The videos of the generalization set were manually labeled as well but kept apart from the model development process to show the potential of generalizability towards “unseen” videos. The process of splitting the data and the subsequent processing in DeepLabCut is visualized in Supplementary Figure S1.

### 2.4 Extraction of x,y coordinates by DeepLabCut

From the video sequence coordinates of body parts (i.e., wrists, elbows, shoulders, hips, knees and ankles) were extracted using markerless motion tracking by the open-source toolbox DeepLabCut (Mathis et al., 2018; Nath et al., 2019). DeepLabCut enables training of a deep neural network using pretrained models with limited training data to track user-defined body parts using transfer learning. DeepLabCut outputs the x,y coordinates of the body part of each frame of the video, as well as the likelihood of prediction (*p*-value). DeepLabCut was run (Version 2.1) using a single NVIDA Tesla K80 GPU platform *via* Microsoft Azure’s cloud with a “Data science Virtual Machine—Windows 2019)” blueprint. The conda environment (for GPU provided by DeepLabCut) was used within a Jupyter notebook. Models were trained using an available residual neural network with 101 layers (ResNet-101) weights pretrained on the MPII Human pose dataset (Andriluka et al., 2014a; Andriluka et al., 2014b; Insafutdinov et al., 2016) as initial weights. For each video, the body parts (i.e., wrists, elbows, shoulders, hips, knees and ankles) of 20 frames were manually labeled. Frames for labeling were automatically selected by DeepLabCut using k-means clustering to select frames with a variety of postures within the datasets. The labeled frames of the development set were randomly split into training and test sets (95% training dataset, 5% test dataset). Training was performed using the default settings of DeepLabcut, e.g., shuffle is true With the dataset to assess tracking accuracy, up to 400,000 iterations were trained. The graphs of cross-entropy loss were inspected to determine convergence and define the minimal training iterations needed for the dataset.

### 2.5 Evaluation of tracking accuracy

All models were evaluated against their own dataset, i.e., test and train error and towards the generalization data set, i.e., generalization error (Supplementary Figure S1). The model evaluation was performed within DeepLabCut by calculation of the Euclidean distance for x,y coordinates (i.e., manually labeled *versus* predicted by the model). The mean of Euclidean distances (across all body points and frames) was taken as the mean absolute error (MAE). MAEs were calculated with and without a p-cutoff of 0.8 (i.e., leaving predictions out with a low likelihood to be correctly identified by the model, e.g., due to occlusion of body parts).

### 2.6 Clinical scores

The original videos were scored by three raters using the DIS (Monbaliu et al., 2012). The DIS evaluates 12 body regions (eyes, mouth, neck, trunk, right and left arm proximal, right and left arm distal, right and left leg proximal, and right and left leg distal) during rest and activity. For our aim, only the videos recorded during rest (sitting in a comfort position, in all cases within their own wheelchair) and lying supine on a mat on the floor were used. Within these items, the proximal lower extremity (during lying) and proximal upper extremity (during sitting) are scored according to the DIS protocol. The amplitude (percentage of range) of dystonia was used within our approach (scoring: 0, 1, 2, 3, 4). (i.e., dystonia leg and dystonia arm). A percentage score was calculated by dividing the individual score by the maximum possible score on the corresponding item (leading to percentage scales of 0, 0.25, 0.50, 0.75 and 1). Three raters (two pediatric therapists and one medical student), all trained to score the DIS, scored different videos, with some overlap (maximal two different raters for one video). The scores of Raters 2 and 3 were collected during the IDYS trial. Each of them scored half of the videos of the whole trial. The same rater always assessed all three time points (baseline, 3-month follow-up and 12-month follow-up) in an individual participant. Rater 1 scored all videos from baseline and 12-month follow-up to compare model-rater accuracy towards inter-rater accuracy.

To allow comparison between inter-rater to model-rater accuracy the mean absolute error (MAE) between Rater 1 and Rater 2, and Rater 1 and Rater 3, respectively, were calculated.

### 2.7 Dataset x,y coordinates for prediction models

For training of the prediction models, the aim was to have as precise coordinates as possible. Therefore, the videos and manual labels (20 manual labels per video) from the generalization set, which were primarily left out in the training process to assess generalization of tracking, were also added to the training set to increase tracking accuracy for the whole dataset. All models were trained up to a minimum of 200,000 iterations with a batch size of one within this step. In addition, movies were created overlaying the stick figures on the original video. These overlay movies were inspected one by one. Additional manual labels were added, and the model was re-trained with these additional labels for all videos where it was deemed necessary. This was the case in 11 videos (especially for children lying or moving towards lateral position, children with wide clothes on—covering the joints and participants with hips flexed more than 90° with knees covering hips).

### 2.8 Engineering of movement and posture features

**FIGURE 1:**
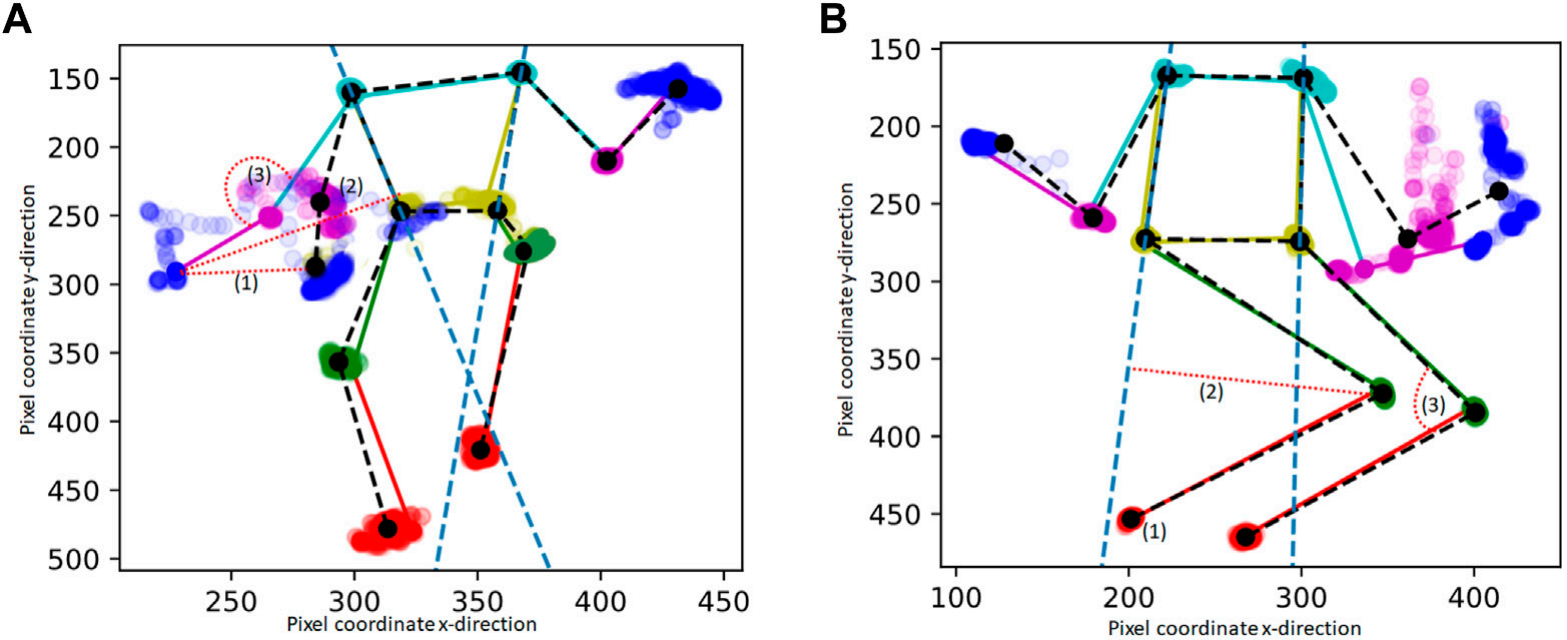
Example of extracted pixel coordinates of one stick figure movie during sitting **(A)** and lying **(B)** “in rest” : The mean stick figure of the videos is plotted in black, in color one example frame is provides, including also the dots of the body points of the whole video. The features are calculated for the left and right wrist and elbow within the sitting videos **(A)** and for the left and right ankle and knee within the lying videos **(B)**. (1), (2), (3) are the features that are extracted from each frame. The features are indicated by a red dotted line: (1) The pixel distance to the average of the body part position in the entire video (distance-to-middle-point), (2) The distance to the line drawn from the shoulder to the hip (the bodyline is indicated with a blue dashed line) (3) The angle of a joint.

Movement and posture features were based on the clinical definition of dystonia to capture movement and postures from the stick figures (x, y coordinates). For the 11 out of 94 videos for the lying position with a shorter video length than 500 frames (i.e. 20 s), the data was extrapolated by adding the existing frames (x,y coordinates) until the length of 500 frames was reached. For each frame, the following three basic features of posture from the extracted x,y coordinates of the body points were computed: (1) distance-to-middle-point, (2) distance-to-line and (3) joint-angle (Figure 1). These features were calculated by: (1) Distance-to-middle-point: the pixel distance to the average of the body part position in the entire video. For sitting videos, the distance-to-middle-point was calculated for wrist, elbow, and shoulder coordinates (Figure 1A), and for lying videos, the distance-to-middle-point was calculated for ankle, knee, and hip coordinates (Figure 1B). (2) Distance-to-line: A “line” was drawn from the shoulder to the hip. For lying videos, the distance-to-line was calculated for ankle and knee coordinates (Figure 1A), and for sitting videos, the distance-to-line was calculated for wrist and elbow coordinates (Figure 1B). (3) Joint angle: For sitting videos, the joint angle was calculated for the elbow joint using the wrist, elbow, and shoulder coordinates (Figure 1A), and for lying videos, the joint angles were calculated for the knee joint using the ankle, knee, and hip coordinates (Figure 1B). Note that the joint angle is not the real joint angle but includes some projection error due to the camera angle, which was not standardized during video collection.

For each feature, the frames were divided into 10 equally sized time windows (2 s). As input for the machine learning model, the median value in those windows was used to represent the distribution of the feature over the entire video in a size-10 vector. Because the resulting set of features is symmetric with respect to the vertical axis of the body, the features and corresponding scores from the two sides of the body were treated as two samples.

### 2.9 Machine learning, evaluation of prediction models

To deal with the expected disagreement between raters, a separate model for each rater to predict arm and leg dystonia was trained from the extracted movement and posture features. The different resulting models were also evaluated using data from the same rater.

With the extracted movement and posture features and the clinical scoring, a random forest regression model was trained. The RandomForestRegressor from scikit-learn (Pedregosa et al., 2011) was used, with standard settings (i.e., number of estimators = 100; criterion is squared error and without a maximum depth set).

To address the small number of samples per rater, 5-fold cross-validation was applied. The data were split into five folds (each containing approximately 20 samples). Data from a single patient was always assigned to a single fold using GroupKFold from scikit-learn (Pedregosa et al., 2011). In addition, the two samples created from a single video (i.e., left and right for the arm and leg, respectively) were always put in same folds. A different model was trained in each iteration (five in total), leaving a different fold out each time used to test the model.

As evaluation metrics the MAEs were computed based on model predictions *versus* clinical human scores on the left-out folds. Model-rater accuracy for each rater was expressed as MAE ±standard deviation (SD).

**FIGURE 2:**
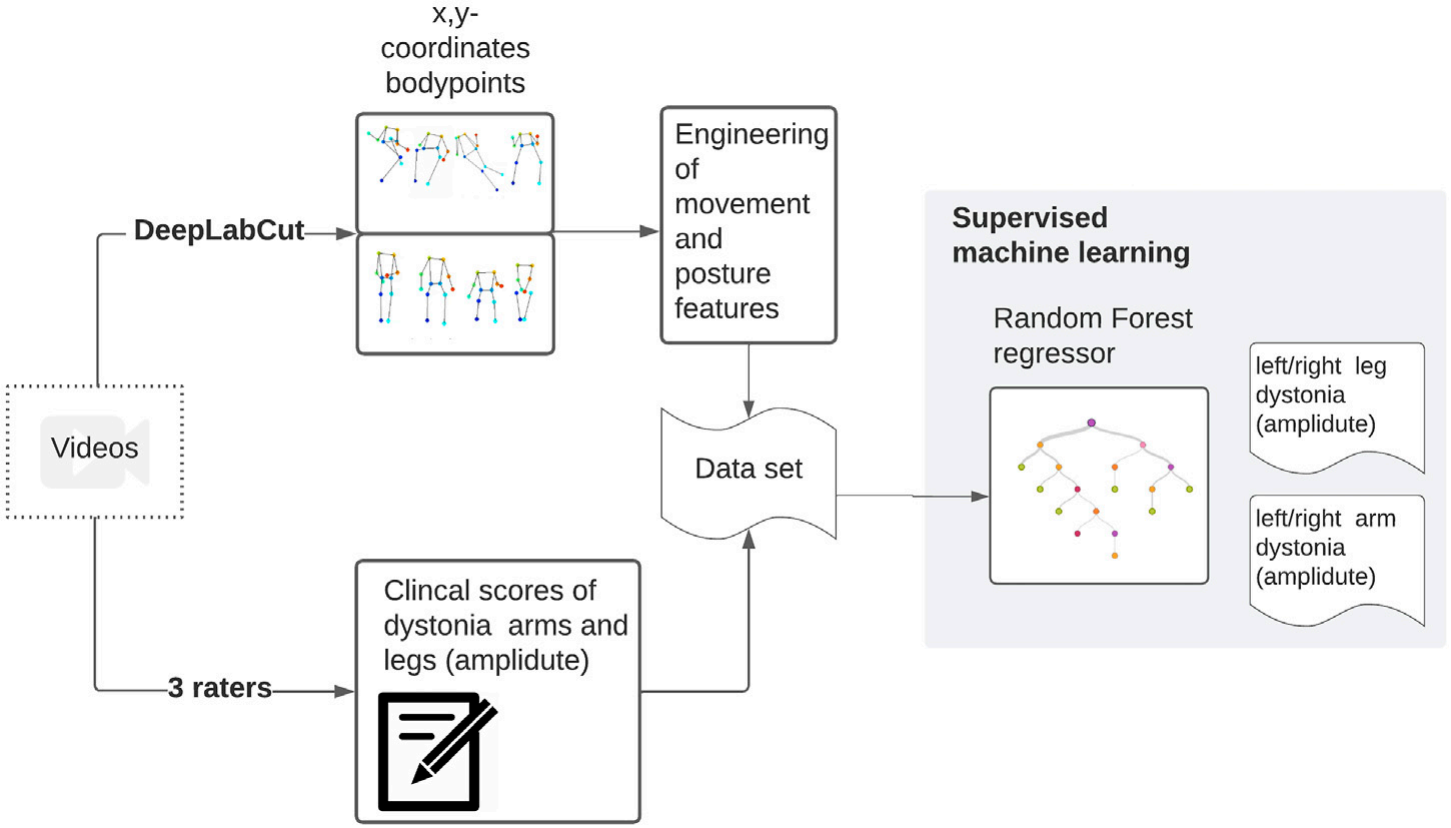
Videos were collected within a clinical trial. These videos were scored by three raters. From these videos, stick figures have been extracted. From the x,y coordinates of the body points, meaningful movement and position features are calculated and used to train a random forest regressor towards the clinical scores.

Confusion matrices were plotted to allow visual comparison for both the inter-rater and model-rater accuracy. The code that was used to perform the analysis is available online (van de Ven et al., 2021). In Figure 2, the whole dataflow is summarized.

## 3 Results

### 3.1 Tracking accuracy

Concerning tracking accuracy within the test set, MAE decreased from 10.09 (1 labeled frame) to 4.49 pixels (20 additionally labeled frames per video) (Table 1). Applying a *p*-value cutoff of 0.8 did not significantly affect the error. The lowest MAEs were reached with the model with 15–20 additional labeled frames per video. Within the generalization set, MAE decreased from 107.04 (no labeled frame—i.e., pretrained model only) to 33.18 pixels (20 labeled frames) (Table 1). Applying a p-cutoff within the generalization set improved the MAE towards 19.88 pixels (Table 1). The x,y coordinates extracted by DeepLabCut are available online, as well as stick figure movies to visualize the data (Haberfehlner et al., 2021).

**TABLE 1:** Mean absolute error (MAE) in the development set (training and testing) and in the generalization set, evaluated with and without p-cutoff 0.8.

Labeled frames	Train set	Test set	Test set with p-cutoff 0.8	Generalization set	Generalization set with p-cutoff 0.8
0				107.04 pixels	121.18 pixels
1	1.17 pixels	10.09 pixels	10.23 pixels	37.24 pixels	28.72 pixels
2	1.11 pixels	5.83 pixels	5.83 pixels	39.00 pixels	28.89 pixels
6	1.65 pixels	5.14 pixels	5.06 pixels	32.82 pixels	23.51 pixels
10	1.8 pixels	5.86 pixels	5.18 pixels	34.68 pixels	25.91 pixels
15	2.36 pixels	4.49 pixels	4.48 pixels	36.26 pixels	28.33 pixels
20	2.71 pixels	4.49 pixels	4.48 pixels	33.18 pixels	19.88 pixels

### 3.2 Prediction models

**FIGURE 3:**
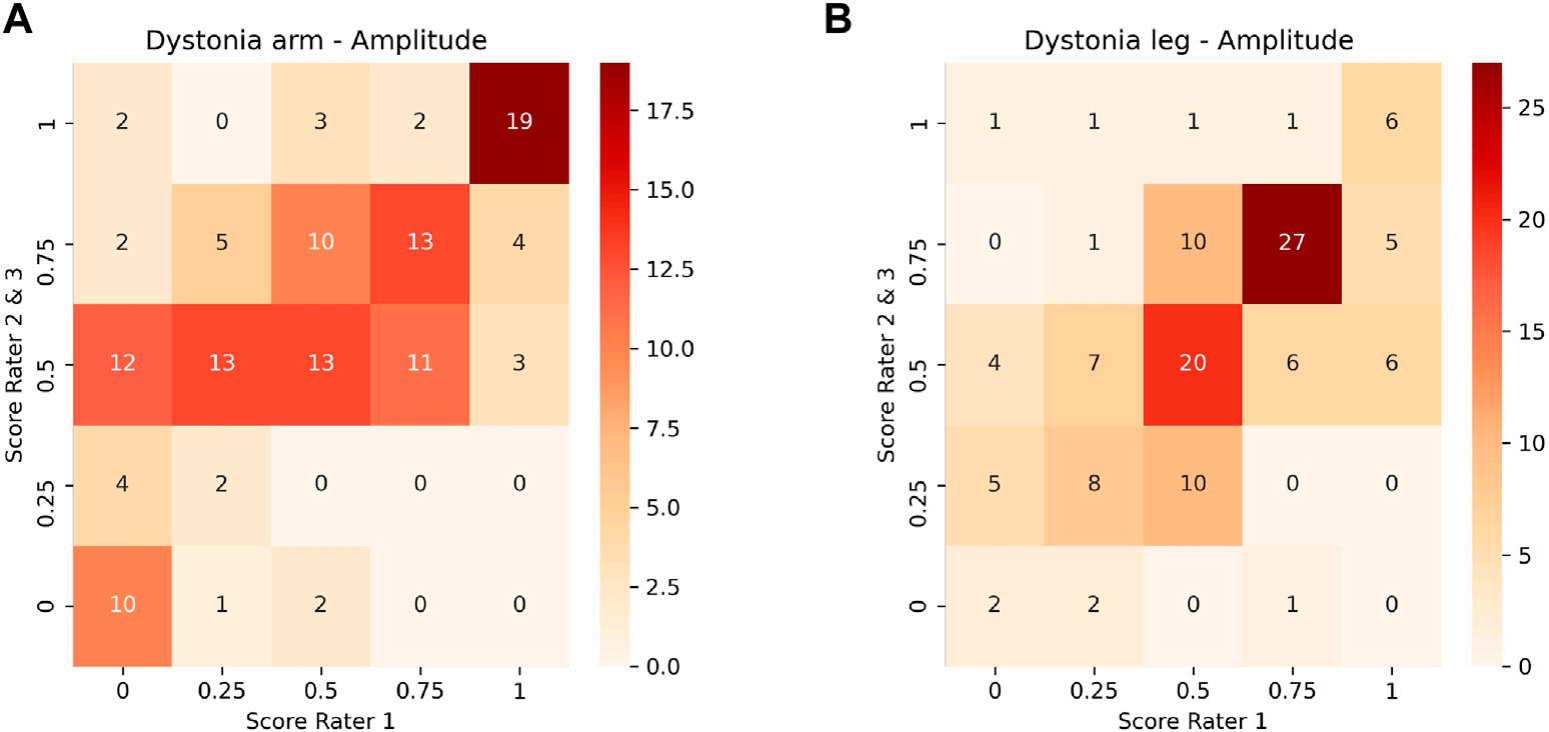
Comparison of scores between Rater 1 and Rater 2 and 3 for the amplitude of dystonia in arm **(A)** and leg **(B)**.

**FIGURE 4:**
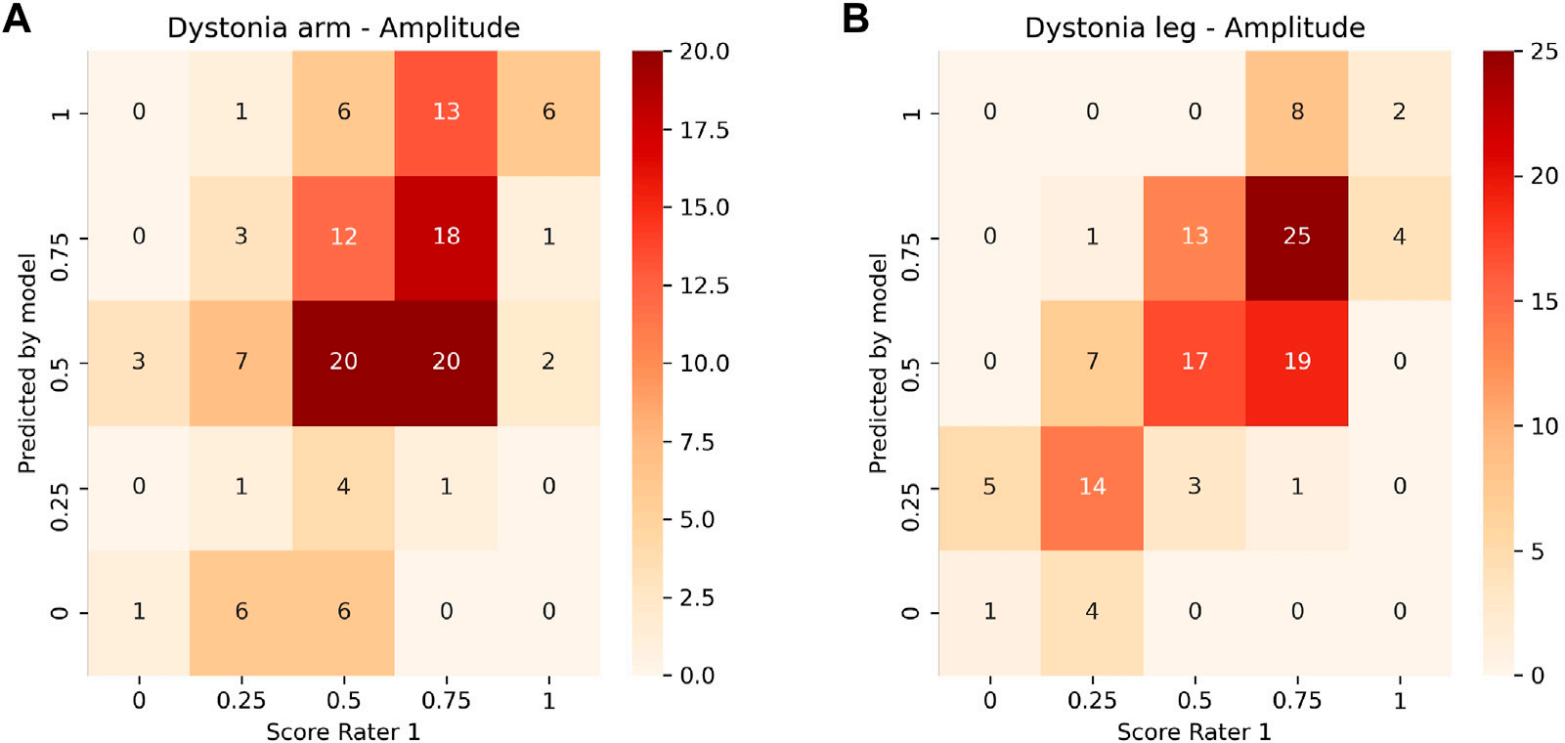
Heat plot of the correlation of the ground truth scoring for Rater 1 towards the predicted scores of the model for the amplitude of arm **(A)** and leg dystonia **(B)**. The values on the diagonal represent a correct prediction.

Inter-rater and model-rater accuracy revealed similar results. Concerning inter-rater accuracy the MAEs ±SD between Rater 1 and Raters 2 and 3 for arm dystonia were 0.21 ± 0.22 and 0.16 ± 0.20 for leg dystonia, respectively (Figure 3). In comparison the model-rater accuracy of the prediction models of Rater 1 reached MAEs ±standard deviation (SD) of 0.21 ± 0.15 for arm dystonia and 0.14 ± 0.10 for leg dystonia. In Figure 4, the “ground truth” of Rater 1 is plotted towards the scores from the model (transformed towards percentage scores). MAEs ± SD for Rater 2 were 0.29 ± 0.19 (arm dystonia) and 0.19 ± 0.17 (leg dystonia) and for Rater 3 were 0.25 ± 0.21 (arm dystonia) and 0.25 ± 0.19 (leg dystonia). The figures for Rater 2 and Rater 3 are provided in the Supplementary Figures S2, S3, respectively and summarized for all raters in Supplementary Figure S4. In all cases, high discrepancies (i.e., differences of >0.5) were rare between the scores predicted by the model and scores given by the human rater (Figure 4 and in Supplementary Figures S2–S4). The detailed results predicted by model *versus* humans are available together with the code online at GitHub in the result section (GitHub—RehabAUmc/modys-video).

## 4 Discussion

This proof-of-concept study is the first time to show the possibility of automatically predicting a dystonia score from common stationary videos, extracting stick figure data (i.e., joint positions) using a machine learning approach. With the use of videos from a common camera, such an approach has the potential to be used within a real-life environment, perhaps even by using smartphone recordings in the future. The results show that the information needed for the model to learn how to score upper and lower leg dystonia at rest is preserved within the extracted stick figures, even with the 2D representation.

The extraction of the required input from videos does not yet work fully automatically. In the current study, it is shown that a tracking error of only 1.5 cm is achieved (compared to human labeling) if 15–20 labeled frames per video are added. Within the current study, DeepLabCut was used to extract coordinates from the videos, as it provides an accessible platform for tuning models towards an own video dataset and assessing the tracking error. For now, using transfer learning based on a pretrained human model in combination with manually added labels is a feasible option to generate input data to further improve our approach for the automated assessment of dystonia.

To reach a fully automatic approach in the future, a generalizable model is needed to extract x,y coordinates from the videos without manually labeling some of the videos first. We have shown that the current dataset (<100 videos) is too small to generate a model that can extract stick figures within an uncommon position from unseen videos within the same dataset. However, the field of human pose estimation is rapidly emerging, with approaches promising even real-time 3D human pose estimation from a single common camera in the near future (Choi et al., 2021). As soon as these approaches become accurate enough to extracted data from pathologic movements and in uncommon situations such as persons in a wheelchair or lying down, stick figures can be easily generated and used as accessible input for the models predicting dystonia scores.

In the current study, conventional supervised machine learning (Random Forest Regressor) was explored. Such algorithms are not suitable to be fed by the whole time series of extracted x,y coordinates. Therefore, movement and posture features are required as input. Within the current study, we engineered features by using the clinical definition of dystonia (i.e., sustained muscle contractions causing abnormal posturing, involuntary and/or distorted voluntary movements) and visually inspecting stick figure data to define features that capture position and movement within the recorded time frame. Features were calculated for each 2-s time window to accommodate for the variability known to occur in dystonia (Sanger, 2006). If more data are available in the future, feature engineering and selection could be performed in a more extensive way as a step within the machine learning process.

Within the trained prediction models to score dystonia at rest, the models trained with the scoring of Rater 1 showed a performance that reached human performance (i.e., by comparing MAEs of inter-rater accuracy and model-rater accuracy). The additional trained models for Rater 2 and Rater 3 showed slightly higher errors. High prediction errors (i.e., >0.5) were rare, and it is necessary to take into account that such a disagreement in scoring obviously also exists between human raters. Within our dataset, the inconsistency could originate from two raters (Rater 2 and 3) having scored the videos spread out over 2 years, whereas Rater 1 scored the videos all in one period (approximately 1 month). To take automated video-based assessment to the next step, it will be important to gather a dataset with high-quality labels. This can be achieved by having multiple (3–10) raters score the same sample, then aggregating the scores using inter-rater statistics into a single gold standard (Dekel and Shamir, 2009). In addition, it will be important to understand the sources of disagreement between raters and inconsistencies within videos scored by the same rater. Models might also improve with a more balanced dataset.

Within the available data, all participants were non-ambulatory (GMFCS IV-V), which is known to be related to higher dystonia scores (Monbaliu et al., 2017b). By adding data from ambulatory children and young adults with dyskinetic CP (GMFCS I-III) or even typically developing subjects, model performance will most likely improve. In addition, a larger dataset will allow to analyze or include factors within the models that possibly affect the prediction such as gender, age, GMCFS and MACS level.

The population within the current study had dystonia as the primary motor disorder, although a mixed presentation of dystonia and spasticity is common in dyskinetic CP (35). The current study was confined to measuring dystonia at rest, where spasticity is not expected to influence the observed dystonia. The largest group of children with dyskinetic CP has limited motor function. However, for children with sufficient motor function, assessment of dystonia during activities (such as reaching, standing) can be of importance (Monbaliu et al., 2012). When assessing dystonia during activity, distinguishing dystonia from spasticity can also possibly become relevant. How to do this, possibly adding EMG, is a topic of future research.

To facilitate the above mentioned possible improvements of the models by pooling the data, we made our data and code open-source available (Haberfehlner et al., 2021; van de Ven et al., 2021). By using markerless motion tracking from 2D videos, historical data and multicenter data can be used to develop a clinically applicable model. Each center can extract x,y coordinates locally, without the need to share videos, and datasets can easily be pooled without privacy issues. Therefore, with considerably larger datasets at hand in the future, automated video-based assessment in dyskinetic CP might also benefit from a deep learning approach to improve the prediction quality of dystonia at rest from stick figures.

## 5 Conclusion

This proof-of-concept study shows the potential of using 2D skeleton stick figures extracted from common videos in a machine learning approach. Even a small data set allows us to train a model that can automatically assess dystonia in the arms and legs in children and young adults with dyskinetic CP in short video sequences with an accuracy comparable to human performance. Expanding the available training data as well as advanced machine learning techniques are the next step to approach the prediction accuracies necessary for clinical use.

## Data Availability

The datasets presented in this study can be found in online repositories. The names of the repository/repositories and accession number(s) can be found below: https://doi.org/10.5281/zenodo.5638470.
