# Supplementary material for: Towards automated video-based assessment of dystonia in dyskinetic cerebral palsy: A novel approach using markerless motion tracking and machine learning
Source: Front Robot AI. 2023 Mar 2;10:1108114. doi: 10.3389/frobt.2023.1108114 (PMC10018017; doi:10.3389/frobt.2023.1108114)
Supplement: Supplementary file 1 [file DataSheet1.PDF]

*Supplementary Material*

# Towards automated video-based assessment of dystonia in dyskinetic cerebral palsy: a novel approach using markerless motion tracking and machine learning

**Helga Haberfehlner*, Shankara S. van de Ven, Sven A. van der Burg, Florian Huber, Sonja Georgievska, Ignazio Aleo, Jaap Harlaar, Laura A. Bonouvrié, Marjolein M. van der Krogt, Annemieke I. Buizer**

*** Correspondence:** Corresponding Author: h.haberfehlner@amsterdamumc.nl

# 1 Supplementary Figures

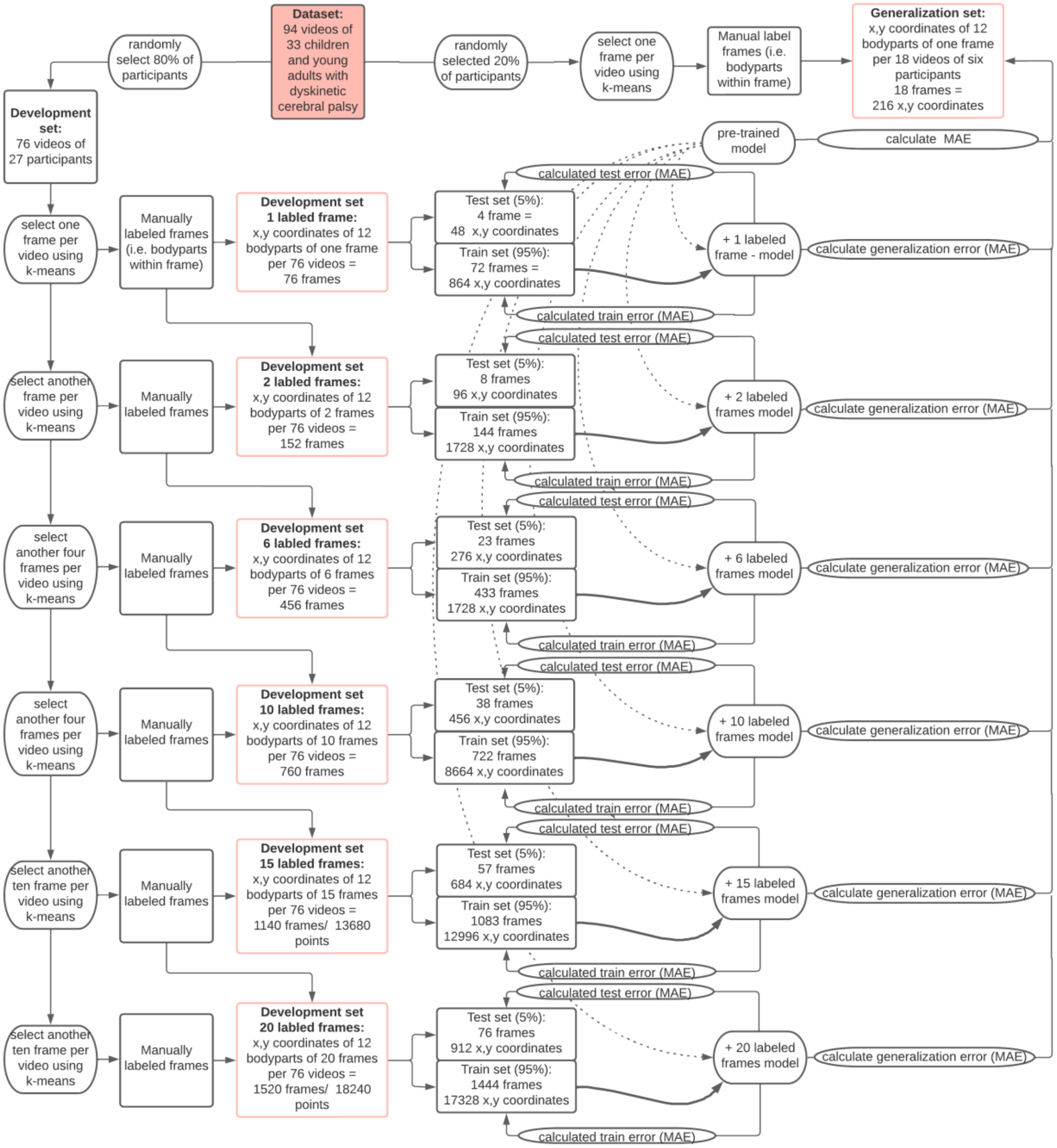


Fig S1. Flow-diagram of steps taken to assess accuracy within our dataset.

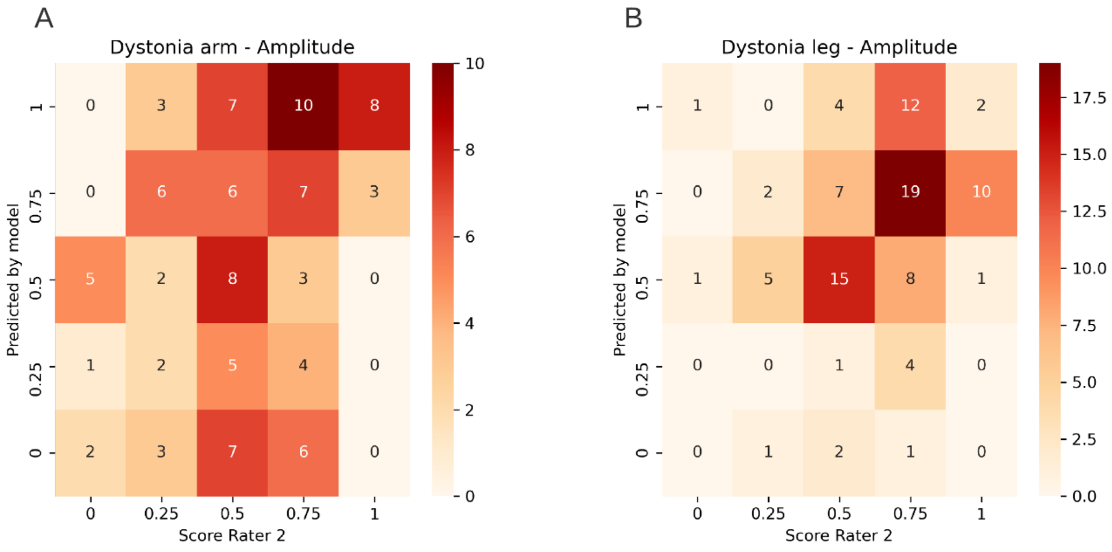


Fig. S2: Heat plot of the correlation of the ground truth scoring of Rater 2 towards the predicted scores of the model for the amplitude of arm (A) and leg dystonia (B).

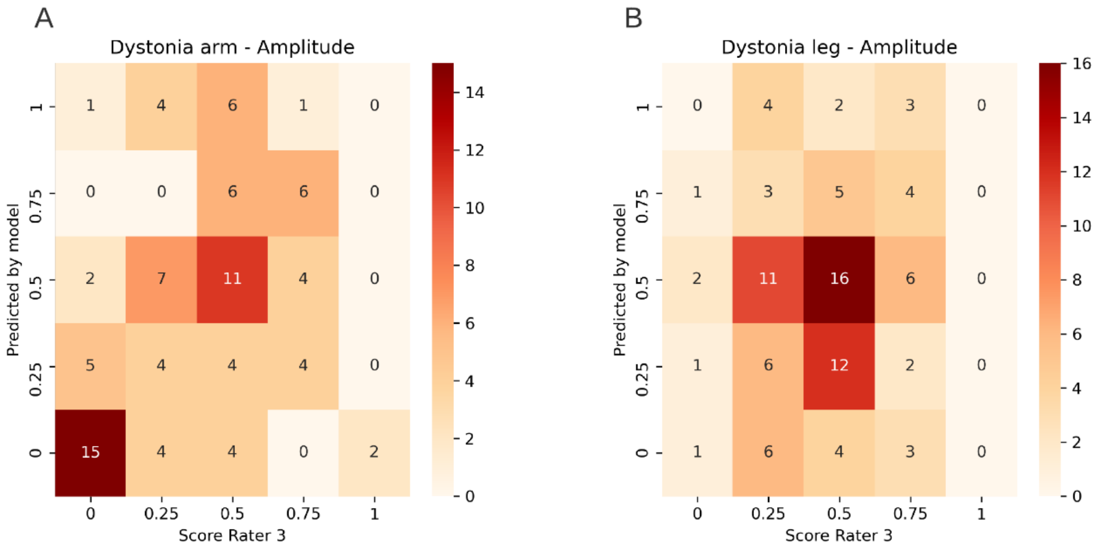


Fig. S3: Heat plot of the correlation of the ground truth scoring of Rater 3 towards the predicted scores of the model for the amplitude of arm (A) and leg dystonia (B).

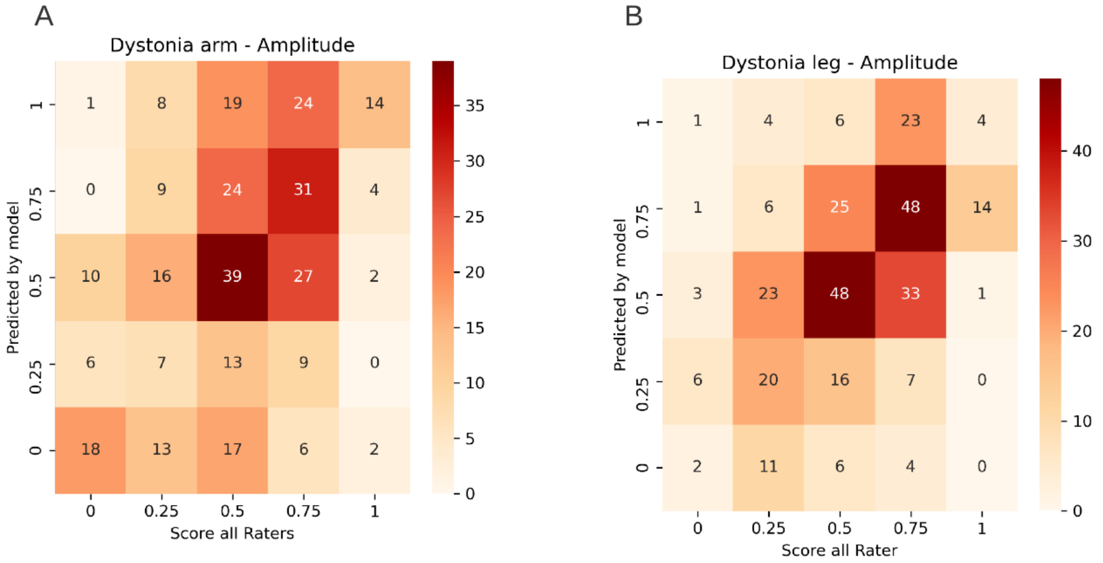


Fig. S4: Heat plot of the correlation of the ground truth scoring (all raters) towards the predicted scores of the models for the amplitude of arm (A) and leg dystonia (B).
